# Predominance of international clone 2 multidrug-resistant *Acinetobacter baumannii* clinical isolates in Thailand: a nationwide study

**DOI:** 10.1186/s12941-021-00424-z

**Published:** 2021-03-20

**Authors:** Piyatip Khuntayaporn, Pohnvipa Kanathum, Jantana Houngsaitong, Preecha Montakantikul, Krit Thirapanmethee, Mullika Traidej Chomnawang

**Affiliations:** 1 Department of Microbiology, Faculty of Pharmacy, Mahidol University, 447 Sri Ayudthya Rd, Rajathevi, Bangkok, 10400 Thailand; 2 Department of Pharmacy, Faculty of Pharmacy, Mahidol University, 447 Sri Ayudthya Rd, Rajathevi, Bangkok, 10400 Thailand

**Keywords:** MDR, Molecular epidemiology, Carbapenemases, MLST, Antimicrobial resistance, Sequence type, *A. baumannii*

## Abstract

**Background:**

*Acinetobacter baumannii* has emerged as one of the common multidrug resistance pathogens causing hospital-acquired infections. This study was conducted to elucidate the distribution of antimicrobial resistance genes in the bacterial population in Thailand. Multidrug-resistant *A. baumannii* (MDR *A. baumannii*) isolates were characterized phenotypically, and the molecular epidemiology of clinical isolates in 11 tertiary hospitals was investigated at a country-wide level.

**Methods:**

A total of 135 nonrepetitive MDR *A. baumannii* isolates collected from tertiary care hospitals across 5 regions of Thailand were examined for antibiotic susceptibility, resistance genes, and sequence types. Multilocus sequence typing (MLST) was performed to characterize the spread of regional lineages.

**Results:**

ST2 belonging to IC2 was the most dominant sequence type in Thailand (65.19%), and to a lesser extent, there was also evidence of the spread of ST164 (10.37%), ST129 (3.70%), ST16 (2.96%), ST98 (2.96%), ST25 (2.96%), ST215 (2.22%), ST338 (1.48%), and ST745 (1.48%). The novel sequence types ST1551, ST1552, ST1553, and ST1557 were also identified in this study. Among these, the *bla*oxa-23 gene was by far the most widespread in MDR *A. baumannii,* while the *bla*oxa-24/40 and *bla*oxa-58 genes appeared to be less dominant in this region. The results demonstrated that the predominant class D carbapenemase was *bla*OXA-23, followed by the class B carbapenemase *bla*NDM-like, while the *mcr*-1 gene was not observed in any isolate. Most of the MDR *A. baumannii* isolates were resistant to ceftazidime (99.23%), gentamicin (91.85%), amikacin (82.96%), and ciprofloxacin (97.78%), while all of them were resistant to carbapenems. The results suggested that colistin could still be effective against MDR *A. baumannii* in this region.

**Conclusion:**

This is the first molecular epidemiological analysis of MDR *A. baumannii* clinical isolates at the national level in Thailand to date. Studies on the clonal relatedness of MDR *A. baumannii* isolates could generate useful data to understand the local epidemiology and international comparisons of nosocomial outbreaks.

**Supplementary Information:**

The online version contains supplementary material available at 10.1186/s12941-021-00424-z.

## Background

Antimicrobial resistance (AMR) has emerged as a silent health threat over the last two decades. It has been estimated that AMR could be the major cause of mortality worldwide by 2050 [1]. Among antimicrobial-resistant bacteria, *Acinetobacter baumannii* is a prominent member of the notorious “ESKAPE” group of pathogens, along with *Enterococcus faecium*, *Staphylococcus aureus*, *Klebsiella pneumoniae*, *Acinetobacter baumannii*, *Pseudomonas aeruginosa*, and *Enterobacter* species [2]. The most common infections caused by *A. baumannii* are related to nosocomial infections, including ventilation-associated pneumonia, skin and soft tissue infections, and bloodstream infections [3, 4]. This organism is well known for its remarkable ability to resist almost all available antibiotics due to its capability of adapting and acquiring resistance genes. Moreover, *A. baumannii* is frequently found to be resistant to a class of last-resort antibiotics, carbapenems, and these strains are known as carbapenem-resistant A. *baumannii* (CRAB). Colistin, an alternative antibiotic, has shown some promising activity against resistant *A. baumannii*. Unfortunately, an increase in the colistin resistance rate has been reported worldwide, along with reports on multidrug-resistant (MDR) and pandrug-resistant (PDR) *A. baumannii* [5]. In Thailand, *A. baumannii* was described as strongly associated with multidrug or carbapenem resistance and has caused nosocomial outbreaks. The presence of MDR *A. baumannii* has been reported in all regions of Thailand since 2000 [6]. According to the annual national antibiogram report 2019, the National Antimicrobial Resistance Surveillance Center, Thailand (NARST), *A. baumannii* complex isolates showed less than 50% susceptibility rates with almost all the currently used antibiotics except colistin [7]. In addition, PDR *A. baumannii* has emerged in regional hospitals in Thailand [8–10].

Molecular techniques have been applied to monitor the epidemiology and evolution of drug-resistant bacteria. Popular techniques include whole-genome sequencing (WGS), multilocus sequence typing (MLST), and pulsed-field gel electrophoresis (PFGE) [5]. PFGE is recognized as the standard method even in this era of sequence-based methods, while WGS is the best method that can provide much information. However, both WGS and PFGE are quite laborious and require specific equipment that is available in only some laboratories. Therefore, MLST is now widely referred to as an additional tool for global epidemiological research that allows the recognition of epidemics and virulence of *A. baumannii* clones and the monitoring of their spread at both the national and international levels. The molecular epidemiology of *A. baumannii* has been extensively studied in outbreaks of infection in many countries worldwide. However, little is known about the clonality and genetic relatedness of MDR *A. baumannii* isolates in Thailand. There were some sporadic reports that focused on a few hospitals in the region. To date, two systemic MLST schemes, namely, the Oxford scheme developed by Bartual et al. and the alternative Pasteur MLST scheme, are now well recognized and acknowledged [11, 12]. These two schemes compare seven housekeeping genes and have three genes in common, namely, *cpn60*, *gltA*, and *recA*. Gaiarsa et al. compared those two schemes and proposed that the Pasteur scheme was more appropriate for population biology and epidemiology studies of *A. baumannii* and related species [13]. Therefore, the Pasteur MLST scheme was referenced in the present study to elucidate the epidemiology of MDR *A. baumannii* in a nationwide study across Thailand. Moreover, the antimicrobial resistance profiles contributing to drugs frequently used in Thailand for MDR *A. baumannii* infections were phenotypically and genetically characterized.

## Materials and methods

### Bacterial collection

*A. baumannii* clinical isolates were collected during 2016–2017 from 11 hospitals in 5 regions of Thailand, including the capital city, central region, northern region, southern region, and northeastern region. Bacteria were preliminarily identified as the genus *Acinetobacter* by microbiological and biochemical methods. All the isolates were genotypically confirmed as *A. baumannii* by the presence of the *bla*oxa-51-like gene [14]. The antibiotic susceptibility profiles of *A. baumannii* in this study were determined by the broth microdilution method according to the Clinical and Laboratory Standards Institute guidelines [15]. MDR *A. baumannii* in this study was defined as nonsusceptible to at least 1 agent in at least 3 antimicrobial categories, including aminoglycosides, fluoroquinolones, carbapenems, cephalosporins, folate pathway inhibitors, penicillins, beta-lactamase inhibitors, tetracyclines, and polymyxins [16]. The samples were stored as glycerol stocks at -80 °C until use.

### Detection of antibiotic resistance genes

The specific primers and PCR conditions for *ndm*-type and *bla*OXA-type carbapenemase detection were used as previously published (Table 1) [17, 18]. *E. coli* ATCC BAA 2469 was purchased from the American Type Culture Collection, USA, and used as a positive control for *ndm*-type carbapenemase. The *bla*oxa-51-like gene, an intrinsic enzyme marker, was used as a marker for the identification of *A. baumannii* according to the study by [14].

**Table 1:** Listed of primers used in this study

Gene	Primers	Product size	References
Sequence typing (ST)			
* cpn60*	ACT GTA CTT GCT CAA GC TTC AGC GAT GAT AAG AAG TGG	405 bp	12
* fusA*	ATC GGT ATT TCT GCK CAC ATY GAT CCA ACA TAC KYT GWA CAC CTT TGT T	633 bp	
* gltA*	AAT TTA CAG TGG CAC ATT AGG TCC C GCA GAG ATA CCA GCA GAG ATA CAC G	483 bp	
* pyrG*	GGT GTT GTT TCA TCA CTA GGW AAA GG ATA AAT GGT AAA GAY TCG ATR TCA CCM A	297 bp	
* recA*	CCT GAA TCT TCY GGT AAA AC GTT TCT GGG CTG CCA AAC ATT AC	372 bp	
* rplB*	GTA GAG CGT ATT GAA TAC GAT CCT AAC C CAC CAC CAC CRT GYG GGT GAT C	330 bp	
* rpoB*	GGCGAAATGGC(AGT)GA(AG)AACCA GA(AG)TC(CT)TCGAAGTTGTAACC	456 bp	
Antimicrobial Resistance Gene (AMG)			
* mcr-1*	CTC GGT CAG TCC GTT TGT TC CCG CAC GAT GTG ACA TTG CT	818 bp	In this study
* bla*ndm-like	CAG CGC AGC TTG TCG TCG CGA AGC TGA GCA	784 bp	17
* bla*oxa-23	GAT CGG ATT GGA GAA CCA GA ATT TCT GAC CGC ATT TCC AT	501 bp	18
* bla*oxa-24/40	GGT TAG TTG GCC CCC TTA AA AGT TGA GCG AAA AGG GGA TT	246 bp	
* bla*oxa-51	TAA TGC TTT GAT CGG CCT TG TGG ATT GCA CTT CAT CTT GG	353 bp	
* bla*oxa-58	AAG TAT TGG GGC TTG TGC TG CCC CTC TGC GCT CTA CAT AC	599 bp	

The *mcr-1* gene from the PubMed database was used as a template to design a set of primers (Table 1). Total genomic DNA of MDR *A. baumannii* was used as a template for the PCR (10 × buffer, 0.2 mM dNTPs, 1 U of DNA polymerase, 0.2 mM each primer, and 2 mM MgCl_2_). The PCR conditions were as follows: 3 min of initial denaturation at 95 °C, followed by 30 cycles of 30 s of denaturation at 95 °C, 30 s of annealing at 61 °C, and 30 s of elongation at 72 °C and a final elongation at 72 °C for 5 min. The PCR products were analyzed by 1% agarose gel electrophoresis, dyed with ethidium bromide and visualized under a UV transilluminator. *E. coli* NCTC 13846 was purchased from the American Type Culture Collection, USA, and used as a positive control.

### Multilocus sequence typing (MLST)

All MDR *A. baumannii* isolates was identified by MLST as described by Diancourt et al. [12]. The MLST profiles were analyzed by using the MLST Pasteur scheme (https://pubmlst.org/abaumannii/). Briefly, total genomic DNA of MDR *A. baumannii* was extracted by the Gentra Puregene Yeast/Bact. Kit (Qiagen, Germany). Seven housekeeping genes (*cpn60, fusA, gltA, pyrG, recA, rplB* and *rpoD*) were amplified by PCR with the recommended conditions and primers (Table 1) using a T100 Thermal Cycler (Bio-Rad, USA). PCR products were sequenced by Bio Basic Inc., Singapore. All DNA sequences were searched for exactly matched locus numbers. Sequence types (STs) were determined by combination of each locus number of each gene.

A new allele number was assigned when the gene was not exactly matched with the existing locus number. PCR products of new loci were amplified and submitted for sequencing to confirm the sequence. New STs were assigned when no STs existed for the combination of all exactly matched loci. All the new alleles and STs information is available at https://pubmlst.org/organisms/ acinetobacter-baumannii [19].

### Clonal complex analysis using GOeBURST

All STs of MDR *A. baumannii* isolates were further analyzed for the clonal complex using Phyloviz 2.0. The program is available at http://www.phyloviz.net/ [20]. A group of STs sharing at least five alleles (DLVs) were allocated to the same clonal complex. All STs in this study were analyzed for MLST relevance with full MSTs.

## Results

### Antimicrobial resistance pattern of MDR *A. baumannii* clinical isolate*s*

**Fig. 1:**
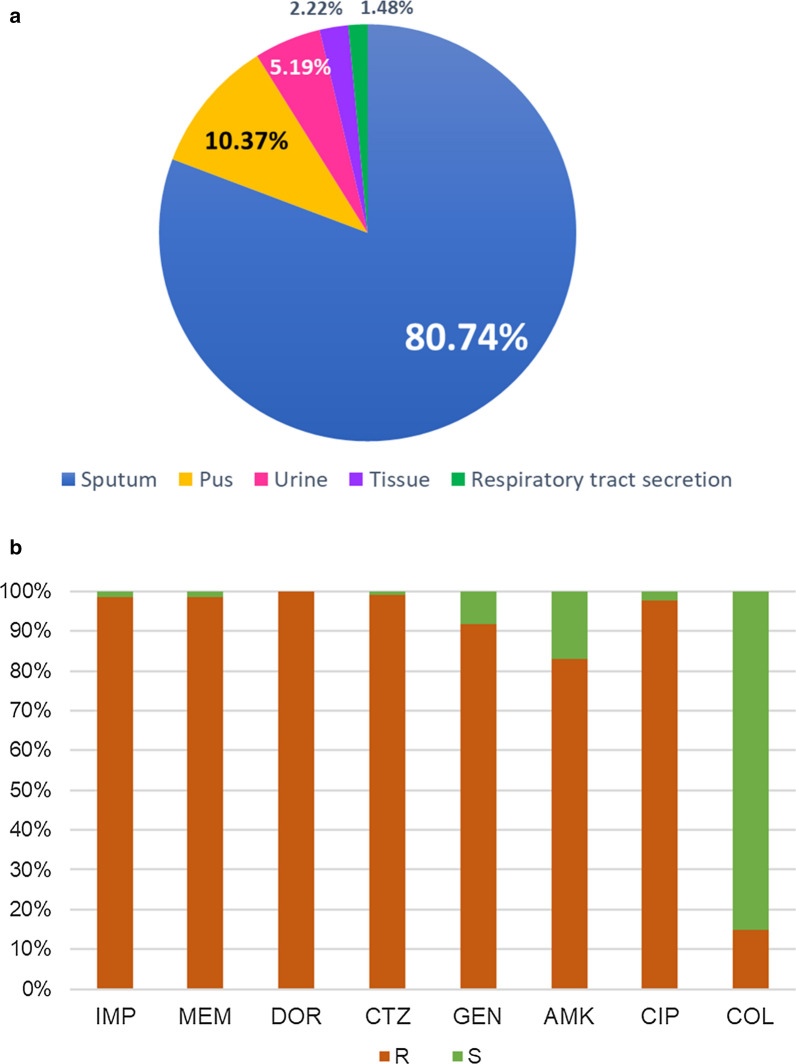
Antimicrobial resistance profile of MDR *A. baumannii.* **a** Sources of specimen. **b** Antibiotic resistance rates. *IMP* imipenem, *MEM* meropenem, *DOR* doripenem, *CTZ* ceftazidime, *GEN* gentamicin, *AMK* amikacin, *CIP* ciprofloxacin, *COL* colistin

A total of 135 nonrepetitive isolates were collected and confirmed to be MDR *A. baumannii* by phenotypic and genotypic methods. The vast majority (109/135, 80.74%) of the isolates were recovered from sputa. The remaining isolates were from pus (14/135, 10.37%), urine (7/135, 5.19%), tissue (3/135, 2.22%), and respiratory tract secretions (2/135, 1.48%) (Fig. 1a). The Acinetobacter isolates were phenotypically identified by microbiological and biochemical methods. The molecular detection of the *bla*oxa-51-like gene revealed a 353-bp band in all clinical isolates, which preliminarily confirmed the identity of the clinical isolates as *A. baumannii*. All clinical isolates were subjected to antimicrobial susceptibility testing with the drugs currently used for *A. baumannii* treatment. Most of the clinical isolates were resistant to ceftazidime (134/135, 99.23%), gentamicin (124/135, 91.85%), amikacin (112/135, 82.96%), ciprofloxacin (132/135, 97.78%), or colistin (20/135, 14.81%), while all the isolates were resistant to carbapenems (imipenem, meropenem, and doripenem). Interestingly, all the isolates showed high resistance to all three carbapenem antibiotics, except two isolates that were still susceptible to both imipenem and meropenem. Notably, 17 out of 135 isolates were resistant to all eight tested antibiotics (Fig. 1b).

### Occurrence of antimicrobial resistance genes in MDR *A. baumannii*

**Fig. 2:**
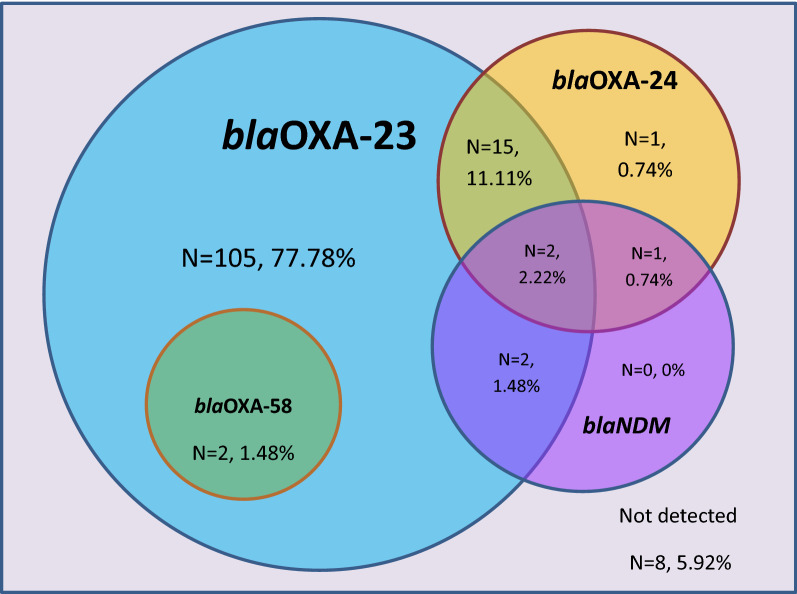
Occurrence of antimicrobial resistance genes among MDR *A. baumannii. *The graphical chart represents the number of antimicrobial genes detected by PCR among all clinical isolates. The majority of class D β-lactamases was *bla*OXA-23 followed by *bla*OXA-24 and *bla*OXA-58 carbapenemases. Some isolates also carry *bla*NDM-type carbapenemases. The overlapped circle represents double or triple existence of the genes

One of the most important carbapenem resistance mechanisms is the production of class D β-lactamases (e.g., oxacillinase; OXA). This study demonstrated that a majority of the class D β-lactamases among MDR *A. baumannii* isolates in Thailand were *bla*oxa-23-like (125/135, 92.59%), followed by *bla*oxa-24-like (20/135, 14.81%), and only two isolates carried the *bla*oxa-58-like gene (1.48%). Notably, MDR *A. baumannii* isolates carrying *bla*oxa-24-like or *bla*oxa-58-like genes also carried the *bla*oxa-23-like gene, indicating the presence of three OXA carbapenemase-encoding genes (including the intrinsic *bla*oxa-51-like gene) in these isolates, while *A. baumannii* ATCC 19606, a standard control strain, exhibited only the intrinsic *bla*oxa-51-like gene (Fig. 2).

In addition, 6 isolates of MDR *A. baumannii* were found to harbor NDM-type carbapenemases collected from two distinct regional hospitals. With an occurrence rate of 4.44%, the data revealed the limited spread of *ndm*-harboring *A. baumannii* isolates in Thailand. Further investigation was performed because colistin is one of the most commonly used drugs in Thailand. Fortunately, the *mcr-1* gene was not detected in any MDR *A. baumannii* isolates in this study. Although colistin resistance phenotypically appeared in some isolates (14.07%), no association with the genotypic presence of the *mcr* gene was identified.

### MLST profile analysis

**Fig. 3:**
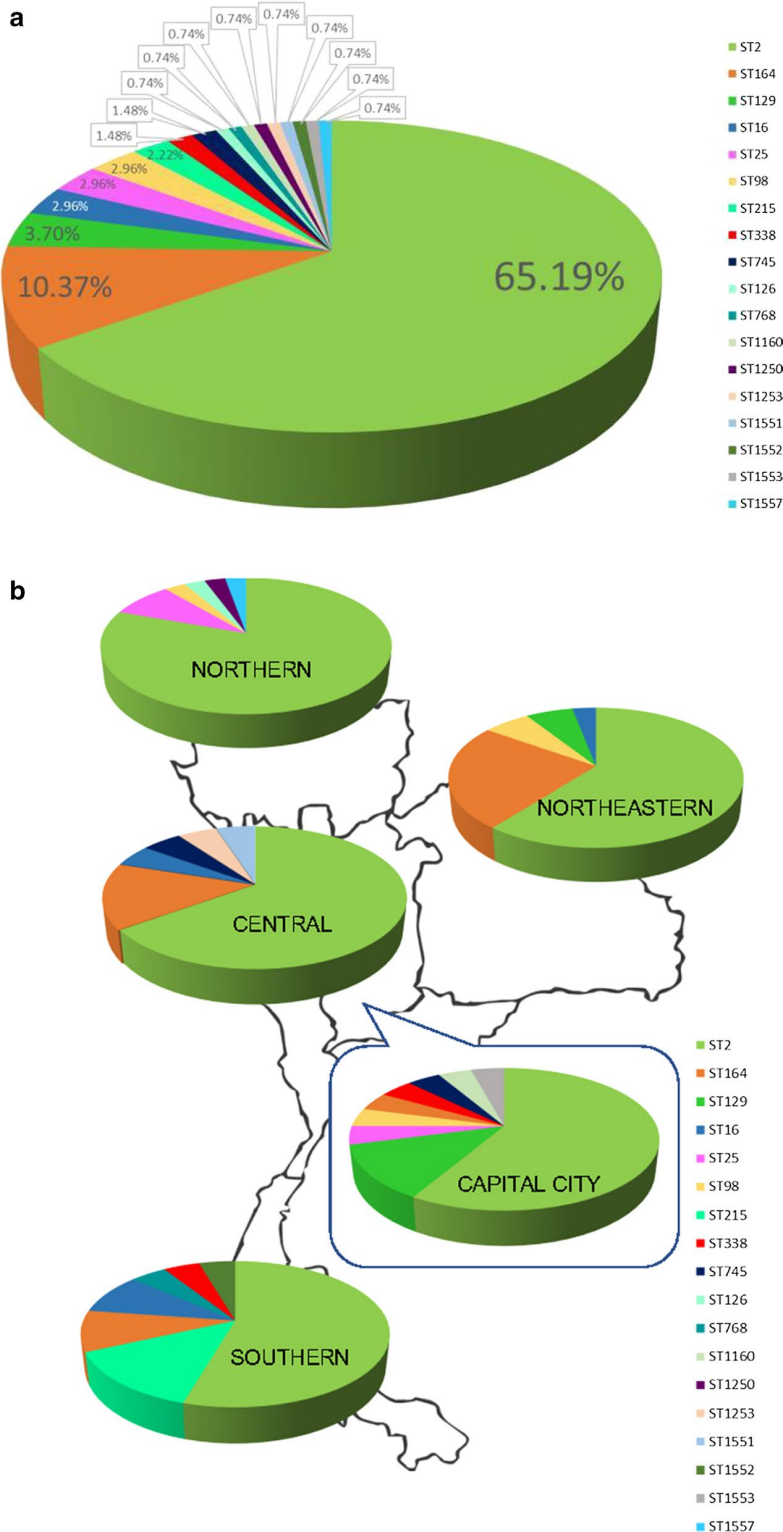
Multi locus sequence typing population analysis. **a** Graph summary of STs in Thailand. **b** The sequence type distribution in each regions of Thailand

In the present study, extensive efforts were made to genetically characterize MDR *A. baumannii* for epidemiological investigation in Thailand. According to the Pasteur MLST scheme, the ST2 or International clone 2 was identified as a dominant clone, with 65.19% (88/135) abundance in this region (Fig. 3a). Another major clone was ST164 (14/135, 10.37%), and other recognized clones were ST129 (5/135, 3.70%), ST16 (4/135, 2.96%), ST98 (4/135, 2.96%), ST25 (4/135, 2.96%), ST215 (3/135, 2.22%), ST338 (2/135, 1.48%), and ST745 (2/135, 1.48%). The remaining five genotypes (e.g., ST768, ST126, ST1160, ST1250, and ST1253) were also identified. Moreover, four new STs of *A. baumannii*, namely, ST1551 (ID5087; MTC0480), ST1552 (ID5088; MTC0904), ST1553 (ID5089; MTC0146) and ST1557 (ID 5104; MTC0709), were discovered in this study. Notably, an MDR *A. baumannii* isolate belonging to ST1553 carried the new allele of the *fusA* gene, which was identified and assigned a new locus number. The newly discovered STs have been deposited in the Acinetobacter MLST database.

Further analysis of the geographic distribution demonstrated different patterns of genetic variants by region (Fig. 3b). ST2 was found to be a dominant clone in all regions of Thailand with varied distribution percentages. The highest percentage of ST2 (80.56%) was observed in the northern region. There were some variations in the ST clones with the second highest percentages between regions. ST129 was ranked second in the capital city, while ST25 and ST215 were ranked second in the northern and southern regions, respectively. In the central and northeastern regions, the second most distributed ST clone was ST164.

**Fig. 4:**
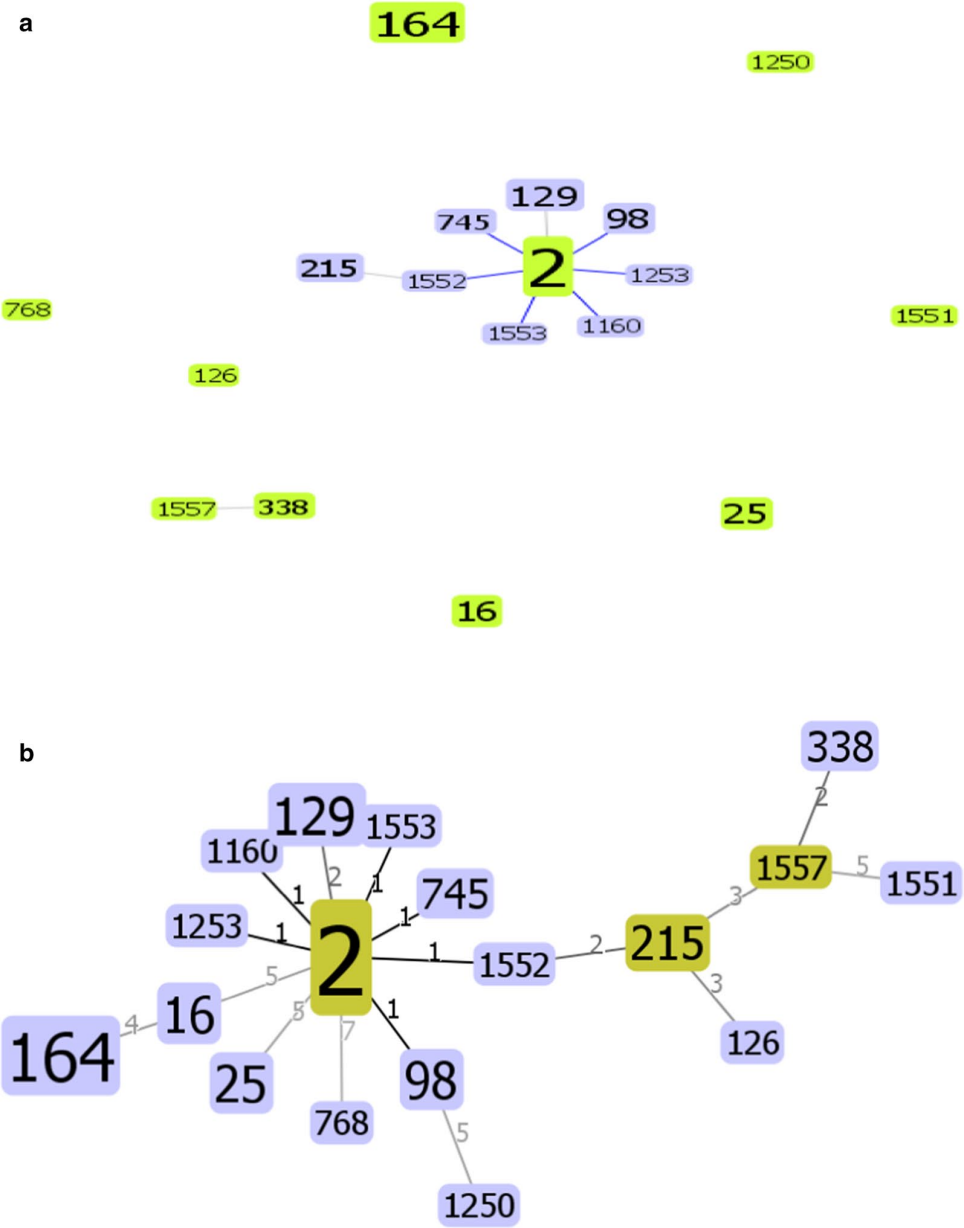
Clonal complex analysis by eBURST. **a** The eBURST analysis showing clonal complexes of MDR *A. baumannii*. Each square signifies the sequence type. The size of each square represents number of isolates, with larger sizes corresponding to higher frequency of occurrence. **b** Relationships among the STs found in this study. Each square signifies to one sequence type. Square size increases correspondingly to the number of isolates of each ST. Each line indicates that the connected squares share the similar alleles. Distance between squares and the numbers are related to the number of allelic mismatches among the corresponding STs

eBURST analysis was performed to assign each ST to the respective clonal complex. The majority of the isolates exhibited a relatively close evolutionary relationship in the phylogenetic tree (Fig. 4). Only one major clonal complex belonging to clonal complex 2 (CC2) was highly prevalent in Thailand. There was no evidence of CC1 being present in this region.

## Discussion

*A. baumannii* is one of the most common emerging nosocomial pathogens, causing significant concern globally. Generally, carbapenems, with the exception of ertapenem, have been the drug of choice for the empirical therapy of *A. baumannii* infections [21]. However, the emergence of CRAB and MDR *A. baumannii* at high frequency in many intensive care units has led to therapeutic failure. Although there is no optimal regimen for elimination of highly resistant *A. baumannii*, colistin is currently recommended for the treatment of serious infections. Other drugs, such as tigecycline and sulbactam, have been reported, but combination therapy is preferable [22]. In this study, all MDR *A. baumannii* isolates were highly resistant to almost all the tested drugs widely used in this region except colistin. The results suggested that colistin could still be used effectively to treat MDR *A. baumannii* infections in Thailand. This study will also raise awareness regarding the monitoring of antibiotic usage with limited resources.

To date, the emergence of carbapenem resistance among MDR *A. baumannii* has been progressively increasing globally. It was clearly demonstrated in this study that the rate of resistance to carbapenems, including imipenem, meropenem, and doripenem, was extremely high (> 90%) among MDR *A. baumannii.* One of the most important carbapenem resistance mechanisms is the production of class D β-lactamases (oxacillinase; OXA). This group of enzymes can hydrolyze oxacillin and third-generation cephalosporins but possesses weak activity against carbapenems [23]. This study demonstrated that the presence of the *bla*oxa-23 gene was closely correlated with carbapenem resistance, and this gene was predominantly carried in MDR *A. baumannii* isolates. Of these genes, the *bla*oxa-23 gene was by far the most widespread in MDR *A. baumannii*, while the *bla*oxa-24/40 and blaoxa-58 genes appeared to be less dominant in this region. The prevalence of the *bla*oxa-23-like gene was found to be relatively high worldwide [5, 24, 25]. Thirapanmethee et al. also reported that the OXA-23-like gene was the most common carbapenemase gene among CRAB clinical isolates in Thailand (68.31%) [26]. In addition, many new enzyme variants of OXA-24/40-like carbapenemases have been reported, including OXA-72. In fact, the *bla*oxa-72 gene was first identified in an *A. baumannii* isolate from Thailand in 2004. Since then, this variant has disseminated in healthcare settings and environments around the world, especially Asia and Southeast Europe, which could be of interest for further investigation [27–30].

Gram-negative pathogens carrying NDM-type beta-lactamases have been discovered worldwide, with high occurrence in India and China [31]. The presence of *bla*NDM-1 demonstrates a pattern of resistance to all beta-lactam agents except monobactams. In Thailand, NDM-type carbapenemase was first detected in Gram-negative pathogens in 2012 [32]. *A. baumannii* carrying NDM-type carbapenemase was later identified in 2018 from the 2013–2014 specimen collection [33]. In the present study, only 4.44% of MDR *A. baumannii* isolates were found to harbor *ndm*-type carbapenemases, and these isolates were collected from two distinct regional hospitals, indicating the limited distribution of this gene type in Thailand.

Colistin is an antibiotic that is highly recommended for use against drug-resistant bacteria. Mobile colistin resistance (MCR-1) was discovered in *Escherichia coli* in 2015 [34]. Since then, many *mcr* gene families (*mcr*-1, -2, -3, -4, -5, -6, -7, -8, -9) have been sporadically reported in Gram-negative bacteria [34–38]. This gene family was also recognized as the latest threat in the antibiotic era since the *mcr* gene was found on transferable plasmids. Fortunately, the *mcr-1* gene was not detected in any MDR *A. baumannii* isolates in this study. Although colistin resistance phenotypically appeared in some isolates (14.07%), no association with the genotypic presence of the *mcr* gene was identified. This could be explained by other colistin resistance mechanisms reported in MDR *A. baumannii,* namely, loss of lipopolysaccharide (LPS) production and mutation of the PmrAB system [39].

To date, a total of 1395 STs have been submitted to the Acinetobacter MLST database [40]. In the present study, extensive efforts were made to genetically characterize MDR *A. baumannii* by MLST analysis for epidemiological investigation in Thailand. MLST has been extensively utilized for genotyping bacteria and allows this genetic information to be placed in a global context. According to the Pasteur MLST scheme, the ST2 or International clone 2 was found to be a dominant clone in all regions of Thailand. A total of 18 ST genotypes played a crucial role in nosocomial infection and spread in Thailand. Moreover, four new STs of *A. baumannii* (ST1551, ST1552, ST1553, and ST1557) were discovered in this study. ST2 has circulated intensively in the rest of the world, including Thailand, as indicated in this study [41, 42]. ST164, ST129, ST25, and ST215 were the common STs in our local settings, as each of them was found in different regions of Thailand. In 2006, a single ST2 isolate obtained from patients hospitalized in intensive care units in Thailand was identified [25]. According to the Acinetobacter MLST database, some Thai ST2 clones and an ST215 *A. baumannii* clone have been present in the database since 2010, indicating local distribution over time [36]. Our previous work demonstrated that most carbapenem-resistant *Acinetobacter baumannii* isolates (CRAB) in Thailand were ST2 clones, but some belonged to ST25, ST98, ST129, ST164, ST215, ST338, and ST745 [26]. In Asia, ST2 has been reported as the most prevalent sequence type of CRAB in China and Lebanon [24, 43, 44]. Sequence typing of *A. baumannii* distributed in Thailand was conducted in a study of 23 samples from a single hospital setting using the Oxford MLST scheme [45]. It was reported that ST195, which belongs to the clonal cluster of IC2, was the dominant clone, followed by ST542. In 2013, the Asian Network for Surveillance of Resistant Pathogens (ANSORP) surveillance study, which randomly selected 30 Thai hospital-associated pneumonia isolates, revealed a high prevalence of ST195, along with some amounts of ST92, ST346, ST88, ST365, ST395, ST208, ST398, and ST399 [46]. However, our study focused on the clonal relatedness of MDR *A. baumannii* using the Pasteur MLST scheme. ST2 classified in IC2 was identified as a dominant clone among MDR *A. baumannii*. Other major clones were incomparable with the previous study since the difference scheme of MLST was assigned.

eBURST analysis was used to analyze the MLST data to determine the evolutionary relationships among the isolates. According to the eBURST analysis, most MDR *A. baumannii* isolates belonged to major clonal complex 2 (CC2), indicating a single dominant lineage in Thailand. Although CC1 and CC2 have been identified as the key clonal lineages globally, no isolates belonging to CC1 were detected in this study. CC2 was by far the most abundant *A. baumannii* clone, with a broad international distribution over many continents [26, 47–50]. CC2 in the Pasteur scheme corresponds to CC92 in the Oxford scheme and international clone II, as previously identified [42]. The majority of MDR *A. baumannii* clones in Thailand belonged to the dominant clonal complex 2, which was similar to the results for other neighboring countries in this region. Tada et al. suggested that ST2 MDR *A. baumannii* isolates were mainly spreading in medical settings in Myanmar [48]. In Malaysia, most of the isolates were grouped under CC92 in the Oxford scheme comparable to CC2 in the Pasteur scheme [51]. Schultz et al. reported that the majority of the CRAB isolates causing ventilator-associated pneumonia (VAP) in Vietnamese ICUs during 2009–2012 were oxa23-positive global clone GC2 [52].

## Conclusion

In summary, our study reported the relative prevalence of MDR *A. baumannii* isolates that were genetically diverse, belonging to 18 distinct STs. The majority of the MLST genotyped isolates in this study fell into one of seven genotypes within a single clonal complex known as IC2. The predominance of IC2 in Thailand was consistent with the high distribution of this clone worldwide. Notably, four new MLST genotypes were discovered in Thailand. The predominant class D carbapenemase was *bla*OXA-23-like, followed by the class B carbapenemase *bla*NDM-like. Fortunately, none of the MDR *A. baumannii* isolates carried the *mcr-1* gene. The present study population was unique and represented the nationwide characterized collection of MDR *A. baumannii* isolates in Thailand reported to date. The data provide knowledge for the evolutionary study and international comparisons of nosocomial outbreaks in the future (Additional file 1).

## Supplementary Information

**Additional file 1.**

## Abbreviations

AMR: Antimicrobial resistance
WGS: Whole genome sequencing
MLST: Multilocus sequence typing
PFGE: Pulse field gel electrophoresis
IMP: Imipenem
MER: Meropenem
DOR: Doripenem
CTZ: Ceftazidime
GEN: Gentamicin
AMK: Amikacin
CIP: Ciprofloxacin
COL: Colistin
*bla*: Beta-lactamase
NDM: New delhi metallo-beta-lactamase
MCR: Mobilized colistin resistance
OXA: Oxacillinase
MDR: Multidrug resistant
PDR: Pan-drug resistant
CRAB: Carbapenem resistant-*Acinetobacter baumannii*
ST: Sequence type
IC: International clone
CC: Clonal complex
GC: Global clone
