# Supplementary material for: Predominance of international clone 2 multidrug-resistant Acinetobacter baumannii clinical isolates in Thailand: a nationwide study
Source: Ann Clin Microbiol Antimicrob. 2021 Mar 20;20:19. doi: 10.1186/s12941-021-00424-z (PMC7980754; doi:10.1186/s12941-021-00424-z)
Supplement: Supplementary file 1 — Additional file 1. [file 12941_2021_424_MOESM1_ESM.docx]

| **No.** | **Culture code** | **Source of Isolation** | **ST** | **Allele ID** | | | | | | | **Antimicrobial Sensitivity** | | | | | | | | ***blaNDM-*like** | ***mcr-1*** | ***blaOXA*** |
| --- | --- | --- | --- | --- | --- | --- | --- | --- | --- | --- | --- | --- | --- | --- | --- | --- | --- | --- | --- | --- | --- |
|  |  |  |  | ***cpn60*** | ***fusA*** | ***gltA*** | ***pyrG*** | ***recA*** | ***rplB*** | ***rpoB*** | **IMP** | **MER** | **DOR** | **CTZ** | **GEN** | **AMK** | **CIP** | **COL** |  |  |  |
| 1 | MTCX104 | Sputum | 2 | 2 | 2 | 2 | 2 | 2 | 2 | 2 | R | R | R | R | R | R | R | S | + | - | 23, 24 |
| 2 | MTCX106 | Sputum | 338 | 8 | 5 | 5 | 26 | 13 | 1 | 2 | R | R | R | R | R | R | R | S | + | - | 23, 24 |
| 3 | MTCX108 | Sputum | 164 | 40 | 3 | 7 | 2 | 40 | 4 | 4 | R | R | R | R | R | S | R | S | - | - | 23 |
| 4 | MTCX110 | Urine | 2 | 2 | 2 | 2 | 2 | 2 | 2 | 2 | R | R | R | R | R | R | R | S | - | - | 23 |
| 5 | MTCX111 | Pus | 2 | 2 | 2 | 2 | 2 | 2 | 2 | 2 | R | R | R | R | R | R | R | S | - | - | 23 |
| 6 | MTCX112 | Sputum | 1160 | 2 | 161 | 2 | 2 | 2 | 2 | 2 | R | R | R | R | R | R | R | S | - | - | 23 |
| 7 | MTC0144 | Urine | 25 | 3 | 3 | 2 | 4 | 7 | 2 | 4 | R | R | R | R | R | R | R | S | - | - | - |
| 8 | MTC0145 | Sputum | 2 | 2 | 2 | 2 | 2 | 2 | 2 | 2 | R | R | R | R | R | R | R | S | - | - | 23 |
| 9 | MTC0146 | Sputum | 1553 | 2 | 225 | 2 | 2 | 2 | 2 | 2 | R | R | R | R | R | R | R | S | - | - | 23 |
| 10 | MTC0147 | Sputum | 2 | 2 | 2 | 2 | 2 | 2 | 2 | 2 | R | R | R | R | R | R | R | S | - | - | 23 |
| 11 | MTC0148 | Sputum | 2 | 2 | 2 | 2 | 2 | 2 | 2 | 2 | R | R | R | R | R | R | R | S | - | - | 23 |
| 12 | MTC0150 | Sputum | 2 | 2 | 2 | 2 | 2 | 2 | 2 | 2 | R | R | R | R | R | R | R | S | - | - | 23 |
| 13 | MTC0151 | Sputum | 2 | 2 | 2 | 2 | 2 | 2 | 2 | 2 | R | R | R | R | R | R | R | S | - | - | 23 |
| 14 | MTC0152 | Sputum | 129 | 3 | 2 | 3 | 2 | 2 | 2 | 2 | R | R | R | R | R | R | R | S | - | - | - |
| 15 | MTC0153 | Sputum | 745 | 2 | 2 | 2 | 2 | 2 | 2 | 101 | R | R | R | R | R | R | R | S | - | - | 23 |
| 16 | MTC0154 | Sputum | 98 | 1 | 2 | 2 | 2 | 2 | 2 | 2 | R | R | R | R | R | R | R | S | - | - | 23 |
| 17 | MTC0155 | Sputum | 129 | 3 | 2 | 3 | 2 | 2 | 2 | 2 | R | R | R | R | R | R | R | S | - | - | 23 |
| 18 | MTC0156 | Sputum | 129 | 3 | 2 | 3 | 2 | 2 | 2 | 2 | R | R | R | R | R | R | R | S | - | - | 23 |
| 19 | MTC0157 | Pus | 2 | 2 | 2 | 2 | 2 | 2 | 2 | 2 | R | R | R | R | R | R | R | S | - | - | - |
| 20 | MTC0158 | Sputum | 2 | 2 | 2 | 2 | 2 | 2 | 2 | 2 | R | R | R | R | R | R | R | S | - | - | 23 |
| 21 | MTC0159 | Sputum | 2 | 2 | 2 | 2 | 2 | 2 | 2 | 2 | R | R | R | R | R | R | R | S | - | - | 23 |
| 22 | MTC0163 | Urine | 2 | 2 | 2 | 2 | 2 | 2 | 2 | 2 | R | R | R | R | R | R | R | S | - | - | 23 |
| 23 | MTC0181 | Sputum | 2 | 2 | 2 | 2 | 2 | 2 | 2 | 2 | R | R | R | R | R | R | R | S | - | - | 23, 24 |
| 24 | MTC0202 | Sputum | 2 | 2 | 2 | 2 | 2 | 2 | 2 | 2 | R | R | R | R | R | R | R | S | - | - | 23 |
| 25 | MTC0304 | Sputum | 745 | 2 | 2 | 2 | 2 | 2 | 2 | 101 | R | R | R | R | R | S | R | S | - | - | 23, 24 |
| 26 | MTC0404 | Sputum | 16 | 7 | 7 | 2 | 2 | 8 | 4 | 4 | R | R | R | R | R | R | R | S | - | - | 23, 58 |
| 27 | MTC0405 | Pus | 2 | 2 | 2 | 2 | 2 | 2 | 2 | 2 | R | R | R | R | R | R | R | S | - | - | 23 |
| 28 | MTC0406 | Sputum | 2 | 2 | 2 | 2 | 2 | 2 | 2 | 2 | R | R | R | R | R | R | R | S | - | - | 23 |
| 29 | MTC0408 | RTS | 2 | 2 | 2 | 2 | 2 | 2 | 2 | 2 | R | R | R | R | R | R | R | S | - | - | 23 |
| 30 | MTC0409 | Pus | 2 | 2 | 2 | 2 | 2 | 2 | 2 | 2 | R | R | R | R | R | R | R | S | - | - | 23 |
| 31 | MTC0411 | Sputum | 2 | 2 | 2 | 2 | 2 | 2 | 2 | 2 | R | R | R | R | R | R | R | S | - | - | 23 |
| 32 | MTC0412 | Sputum | 164 | 40 | 3 | 7 | 2 | 40 | 4 | 4 | R | R | R | R | S | S | R | S | - | - | 23 |
| 33 | MTC0413 | Sputum | 2 | 2 | 2 | 2 | 2 | 2 | 2 | 2 | R | R | R | R | R | R | R | S | - | - | 23 |
| 34 | MTC0414 | Sputum | 1253 | 2 | 2 | 2 | 2 | 2 | 2 | 162 | R | R | R | R | R | R | R | S | - | - | 23 |
| 35 | MTC0415 | Sputum | 164 | 40 | 3 | 7 | 2 | 40 | 4 | 4 | R | R | R | R | S | S | R | S | - | - | 23 |
| 36 | MTC0416 | Sputum | 2 | 2 | 2 | 2 | 2 | 2 | 2 | 2 | R | R | R | R | R | R | R | S | - | - | 23 |
| 37 | MTC0418 | Sputum | 2 | 2 | 2 | 2 | 2 | 2 | 2 | 2 | R | R | R | R | R | R | R | S | - | - | 23, 58 |
| 38 | MTC0420 | Sputum | 2 | 2 | 2 | 2 | 2 | 2 | 2 | 2 | R | R | R | R | R | R | R | S | - | - | 23 |
| 39 | MTC0422 | Sputum | 2 | 2 | 2 | 2 | 2 | 2 | 2 | 2 | R | R | R | R | R | R | R | S | - | - | 23 |
| 40 | MTC0424 | Sputum | 2 | 2 | 2 | 2 | 2 | 2 | 2 | 2 | R | R | R | R | R | R | R | S | - | - | 23 |
| 41 | MTC0427 | Pus | 2 | 2 | 2 | 2 | 2 | 2 | 2 | 2 | R | R | R | R | R | R | R | S | - | - | 23 |
| 42 | MTC0428 | Sputum | 2 | 2 | 2 | 2 | 2 | 2 | 2 | 2 | R | R | R | R | R | R | R | S | - | - | 23 |
| 43 | MTC0429 | Sputum | 164 | 40 | 3 | 7 | 2 | 40 | 4 | 4 | R | R | R | R | S | S | R | S | - | - | 23, 24 |
| 44 | MTC0430 | Sputum | 1551 | 8 | 1 | 7 | 3 | 3 | 2 | 3 | R | R | R | R | R | R | R | S | - | - | 23 |
| 45 | MTC0501 | Sputum | 2 | 2 | 2 | 2 | 2 | 2 | 2 | 2 | R | R | R | R | R | R | R | S | - | - | 23 |
| 46 | MTC0503 | Sputum | 25 | 3 | 3 | 2 | 4 | 7 | 2 | 4 | R | R | R | R | R | R | R | S | - | - | 23 |
| 47 | MTC0507 | Sputum | 2 | 2 | 2 | 2 | 2 | 2 | 2 | 2 | R | R | R | R | R | R | R | S | - | - | 23 |
| 48 | MTC0508 | Sputum | 2 | 2 | 2 | 2 | 2 | 2 | 2 | 2 | R | R | R | R | R | R | R | S | - | - | 23 |
| 49 | MTC0511 | Sputum | 2 | 2 | 2 | 2 | 2 | 2 | 2 | 2 | R | R | R | R | R | R | R | S | - | - | 23 |
| 50 | MTC0520 | Sputum | 98 | 1 | 2 | 2 | 2 | 2 | 2 | 2 | R | R | R | R | R | R | R | S | - | - | 23 |
| 51 | MTC0602 | Sputum | 1250 | 1 | 8 | 2 | 6 | 137 | 1 | 158 | R | R | R | S | S | S | R | R | - | - | 23 |
| 52 | MTC0603 | Urine | 25 | 3 | 3 | 2 | 4 | 7 | 2 | 4 | R | R | R | R | S | S | S | R | - | - | 23 |
| 53 | MTC0606 | Sputum | 2 | 2 | 2 | 2 | 2 | 2 | 2 | 2 | R | R | R | R | R | R | R | R | - | - | 23 |
| 54 | MTC0608 | Sputum | 2 | 2 | 2 | 2 | 2 | 2 | 2 | 2 | R | R | R | R | R | R | R | R | - | - | 23 |
| 55 | MTC0609 | Sputum | 2 | 2 | 2 | 2 | 2 | 2 | 2 | 2 | R | R | R | R | R | R | R | R | - | - | 23 |
| 56 | MTC0610 | Sputum | 2 | 2 | 2 | 2 | 2 | 2 | 2 | 2 | R | R | R | R | R | R | R | R | - | - | 23 |
| 57 | MTC0612 | Sputum | 2 | 2 | 2 | 2 | 2 | 2 | 2 | 2 | R | R | R | R | R | R | R | S | - | - | 23 |
| 58 | MTC0614 | Pus | 2 | 2 | 2 | 2 | 2 | 2 | 2 | 2 | R | R | R | R | R | R | R | R | - | - | 23 |
| 59 | MTC0615 | Sputum | 2 | 2 | 2 | 2 | 2 | 2 | 2 | 2 | R | R | R | R | R | R | R | R | - | - | 23 |
| 60 | MTC0617 | Sputum | 2 | 2 | 2 | 2 | 2 | 2 | 2 | 2 | R | R | R | R | R | R | R | R | - | - | 23 |
| 61 | MTC0619 | Sputum | 25 | 3 | 3 | 2 | 4 | 7 | 2 | 4 | R | R | R | R | R | R | R | R | - | - | 23 |
| 62 | MTC0620 | Sputum | 2 | 2 | 2 | 2 | 2 | 2 | 2 | 2 | R | R | R | R | R | R | R | R | - | - | 23 |
| 63 | MTC0623 | Sputum | 2 | 2 | 2 | 2 | 2 | 2 | 2 | 2 | R | R | R | R | R | R | R | R | - | - | 23 |
| 64 | MTC0627 | Sputum | 2 | 2 | 2 | 2 | 2 | 2 | 2 | 2 | R | R | R | R | R | R | R | R | - | - | 23 |
| 65 | MTC0629 | Sputum | 2 | 2 | 2 | 2 | 2 | 2 | 2 | 2 | R | R | R | R | R | R | R | R | - | - | - |
| 66 | MTC0701 | Tissue | 2 | 2 | 2 | 2 | 2 | 2 | 2 | 2 | R | R | R | R | R | R | R | S | - | - | 23, 24 |
| 67 | MTC0702 | Sputum | 2 | 2 | 2 | 2 | 2 | 2 | 2 | 2 | R | R | R | R | R | R | R | S | - | - | 23, 24 |
| 68 | MTC0703 | Sputum | 126 | 3 | 2 | 7 | 2 | 7 | 1 | 3 | **S** | **S** | R | R | R | S | R | S | - | - | 23 |
| 69 | MTC0705 | Sputum | 2 | 2 | 2 | 2 | 2 | 2 | 2 | 2 | R | R | R | R | S | S | R | S | - | - | 23, 24 |
| 70 | MTC0706 | Sputum | 2 | 2 | 2 | 2 | 2 | 2 | 2 | 2 | R | R | R | R | R | S | R | S | - | - | 24 |
| 71 | MTC0707 | Sputum | 2 | 2 | 2 | 2 | 2 | 2 | 2 | 2 | R | R | R | R | R | R | R | S | - | - | 23, 24 |
| 72 | MTC0708 | Sputum | 2 | 2 | 2 | 2 | 2 | 2 | 2 | 2 | R | R | R | R | R | R | R | S | - | - | 23, 24 |
| 73 | MTC0709 | Sputum | 1557 | 8 | 5 | 7 | 2 | 13 | 1 | 2 | R | R | R | R | R | R | S | S | + | - | 24 |
| 74 | MTC0710 | Sputum | 2 | 2 | 2 | 2 | 2 | 2 | 2 | 2 | R | R | R | R | R | R | R | S | - | - | 23 |
| 75 | MTC0712 | Sputum | 2 | 2 | 2 | 2 | 2 | 2 | 2 | 2 | R | R | R | R | R | R | R | S | - | - | 23 |
| 76 | MTC0714 | Sputum | 2 | 2 | 2 | 2 | 2 | 2 | 2 | 2 | R | R | R | R | R | R | R | S | - | - | 23, 24 |
| 77 | MTC0716 | Tissue | 2 | 2 | 2 | 2 | 2 | 2 | 2 | 2 | R | R | R | R | R | R | R | S | + | - | 23 |
| 78 | MTC0718 | Sputum | 2 | 2 | 2 | 2 | 2 | 2 | 2 | 2 | R | R | R | R | R | R | R | S | + | - | 23, 24 |
| 79 | MTC0719 | Sputum | 2 | 2 | 2 | 2 | 2 | 2 | 2 | 2 | R | R | R | R | R | R | R | S | + | - | 23 |
| 80 | MTC0802 | Sputum | 2 | 2 | 2 | 2 | 2 | 2 | 2 | 2 | R | R | R | R | R | R | R | S | - | - | 23 |
| 81 | MTC0807 | Sputum | 2 | 2 | 2 | 2 | 2 | 2 | 2 | 2 | R | R | R | R | R | R | R | S | - | - | 23 |
| 82 | MTC0808 | Sputum | 2 | 2 | 2 | 2 | 2 | 2 | 2 | 2 | R | R | R | R | R | R | R | S | - | - | 23 |
| 83 | MTC0810 | Sputum | 2 | 2 | 2 | 2 | 2 | 2 | 2 | 2 | R | R | R | R | R | R | R | S | - | - | 23 |
| 84 | MTC0811 | Pus | 164 | 40 | 3 | 7 | 2 | 40 | 4 | 4 | R | R | R | R | R | S | R | S | - | - | 23 |
| 85 | MTC0812 | Pus | 2 | 2 | 2 | 2 | 2 | 2 | 2 | 2 | R | R | R | R | R | R | R | S | - | - | 23 |
| 86 | MTC0815 | Sputum | 2 | 2 | 2 | 2 | 2 | 2 | 2 | 2 | R | R | R | R | R | R | R | S | - | - | 23 |
| 87 | MTC0816 | Sputum | 2 | 2 | 2 | 2 | 2 | 2 | 2 | 2 | R | R | R | R | R | S | R | S | - | - | 23 |
| 88 | MTC0818 | Sputum | 2 | 2 | 2 | 2 | 2 | 2 | 2 | 2 | R | R | R | R | R | R | R | S | - | - | 23 |
| 89 | MTC0825 | Sputum | 2 | 2 | 2 | 2 | 2 | 2 | 2 | 2 | R | R | R | R | R | R | R | S | - | - | - |
| 90 | MTC0901 | Tissue | 768 | 22 | 26 | 91 | 18 | 23 | 19 | 47 | R | R | R | R | R | R | R | S | - | - | - |
| 91 | MTC0902 | Pus | 215 | 27 | 7 | 7 | 2 | 2 | 1 | 2 | R | R | R | R | R | R | R | S | - | - | 23 |
| 92 | MTC0904 | Sputum | 1552 | 27 | 2 | 2 | 2 | 2 | 2 | 2 | R | R | R | R | R | R | R | S |  |  | 23 |
| 93 | MTC0905 | Sputum | 215 | 27 | 2 | 7 | 2 | 2 | 1 | 2 | R | R | R | R | R | R | R | S | - | - | 23 |
| 94 | MTC0907 | Sputum | 164 | 40 | 3 | 7 | 2 | 40 | 4 | 4 | R | R | R | R | R | R | R | S | - | - | - |
| 95 | MTC0909 | Sputum | 16 | 7 | 7 | 2 | 2 | 8 | 4 | 4 | R | R | R | R | R | R | R | S | - | - | 23 |
| 96 | MTC0911 | Sputum | 2 | 2 | 2 | 2 | 2 | 2 | 2 | 2 | R | R | R | R | R | R | R | S | - | - | 23 |
| 97 | MTC0912 | Pus | 2 | 2 | 2 | 2 | 2 | 2 | 2 | 2 | R | R | R | R | R | R | R | S | - | - | 23, 24 |
| 98 | MTC0921 | Sputum | 2 | 2 | 2 | 2 | 2 | 2 | 2 | 2 | R | R | R | R | S | S | R | S | - | - | 23 |
| 99 | MTC0922 | Sputum | 16 | 7 | 7 | 2 | 2 | 8 | 4 | 4 | R | R | R | R | R | R | R | S | - | - | 23 |
| 100 | MTC0923 | Sputum | 215 | 27 | 2 | 7 | 2 | 2 | 1 | 2 | R | R | R | R | R | R | R | S | - | - | 23 |
| 101 | MTC0924 | Sputum | 338 | 8 | 5 | 5 | 26 | 13 | 1 | 2 | R | R | R | R | R | R | R | S | - | - | 23 |
| 102 | MTC1102 | Urine | 164 | 40 | 3 | 7 | 2 | 40 | 4 | 4 | R | R | R | R | R | S | R | S | - | - | 23 |
| 103 | MTC1104 | Urine | 164 | 40 | 3 | 7 | 2 | 40 | 4 | 4 | R | R | R | R | S | S | R | S | - | - | 23 |
| 104 | MTC1106 | Pus | 2 | 2 | 2 | 2 | 2 | 2 | 2 | 2 | R | R | R | R | R | R | R | R | - | - | 23 |
| 105 | MTC1107 | Pus | 164 | 40 | 3 | 7 | 2 | 40 | 4 | 4 | R | R | R | R | S | S | R | S | - | - | 23 |
| 106 | MTC1109 | Urine | 164 | 40 | 3 | 7 | 2 | 40 | 4 | 4 | R | R | R | R | R | S | R | S | - | - | 23 |
| 107 | MTC1111 | Pus | 164 | 40 | 3 | 7 | 2 | 40 | 4 | 4 | R | R | R | R | S | S | R | S | - | - | 23 |
| 108 | MTC1112 | Sputum | 2 | 2 | 2 | 2 | 2 | 2 | 2 | 2 | R | R | R | R | R | R | R | S | - | - | 23 |
| 109 | MTC1113 | Sputum | 2 | 2 | 2 | 2 | 2 | 2 | 2 | 2 | R | R | R | R | R | R | R | S | - | - | 23 |
| 110 | MTC1114 | Sputum | 129 | 3 | 2 | 3 | 2 | 2 | 2 | 2 | R | R | R | R | R | R | R | S | - | - | 23 |
| 111 | MTC1115 | Sputum | 129 | 3 | 2 | 3 | 2 | 2 | 2 | 2 | R | R | R | R | R | R | R | S | - | - | 23 |
| 112 | MTC1117 | Sputum | 2 | 2 | 2 | 2 | 2 | 2 | 2 | 2 | R | R | R | R | R | R | R | S | - | - | 23 |
| 113 | MTC1118 | Sputum | 2 | 2 | 2 | 2 | 2 | 2 | 2 | 2 | R | R | R | R | R | S | R | S | - | - | 23 |
| 114 | MTC1119 | Sputum | 16 | 7 | 7 | 2 | 2 | 8 | 4 | 4 | R | R | R | R | R | R | R | S | - | - | 23 |
| 115 | MTC1120 | Sputum | 2 | 2 | 2 | 2 | 2 | 2 | 2 | 2 | R | R | R | R | R | R | R | R | - | - | 23 |
| 116 | MTC1121 | Sputum | 2 | 2 | 2 | 2 | 2 | 2 | 2 | 2 | R | R | R | R | R | R | R | R | - | - | 23 |
| 117 | MTC1122 | Sputum | 164 | 40 | 3 | 7 | 2 | 40 | 4 | 4 | R | R | R | R | S | S | R | S | - | - | 23 |
| 118 | MTC1123 | Sputum | 164 | 40 | 3 | 7 | 2 | 40 | 4 | 4 | R | R | R | R | R | S | R | S | - | - | 23 |
| 119 | MTC1124 | Sputum | 164 | 40 | 3 | 7 | 2 | 40 | 4 | 4 | R | R | R | R | R | S | R | R | - | - | 23 |
| 120 | MTC1125 | Sputum | 2 | 2 | 2 | 2 | 2 | 2 | 2 | 2 | R | R | R | R | R | R | R | R | - | - | 23 |
| 121 | MTC1127 | Sputum | 98 | 1 | 2 | 2 | 2 | 2 | 2 | 2 | R | R | R | R | R | R | R | S | - | - | 23 |
| 122 | MTC1128 | Sputum | 98 | 1 | 2 | 2 | 2 | 2 | 2 | 2 | **S** | **S** | R | R | R | S | S | S | - | - | 23 |
| 123 | MTC1130 | Sputum | 2 | 2 | 2 | 2 | 2 | 2 | 2 | 2 | R | R | R | R | R | R | R | R | - | - | 23 |
| 124 | MTC1305 | RTS | 2 | 2 | 2 | 2 | 2 | 2 | 2 | 2 | R | R | R | R | R | R | R | S | - | - | 23 |
| 125 | MTC1306 | Sputum | 2 | 2 | 2 | 2 | 2 | 2 | 2 | 2 | R | R | R | R | R | R | R | S | - | - | 23 |
| 126 | MTC1310 | Sputum | 2 | 2 | 2 | 2 | 2 | 2 | 2 | 2 | R | R | R | R | R | R | R | S | - | - | 23 |
| 127 | MTC1313 | Sputum | 2 | 2 | 2 | 2 | 2 | 2 | 2 | 2 | R | R | R | R | R | R | R | S | - | - | 23 |
| 128 | MTC1314 | Sputum | 2 | 2 | 2 | 2 | 2 | 2 | 2 | 2 | R | R | R | R | R | R | R | S | - | - | 23 |
| 129 | MTC1318 | Sputum | 2 | 2 | 2 | 2 | 2 | 2 | 2 | 2 | R | R | R | R | R | R | R | S | - | - | 23, 24 |
| 130 | MTC1320 | Sputum | 2 | 2 | 2 | 2 | 2 | 2 | 2 | 2 | R | R | R | R | R | R | R | S | - | - | 23, 24 |
| 131 | MTC1322 | Pus | 2 | 2 | 2 | 2 | 2 | 2 | 2 | 2 | R | R | R | R | R | R | R | S | - | - | 23 |
| 132 | MTC1325 | Sputum | 2 | 2 | 2 | 2 | 2 | 2 | 2 | 2 | R | R | R | R | R | R | R | S | - | - | - |
| 133 | MTC1330 | Sputum | 2 | 2 | 2 | 2 | 2 | 2 | 2 | 2 | R | R | R | R | R | R | R | S | - | - | 23, 24 |
| 134 | MTC1331 | Sputum | 2 | 2 | 2 | 2 | 2 | 2 | 2 | 2 | R | R | R | R | R | R | R | S | - | - | 23, 24 |
| 135 | MTC0516 | Sputum | 2 | 2 | 2 | 2 | 2 | 2 | 2 | 2 | R | R | R | R | R | R | R | S | - | - | 23, 24 |

**Abbreviation:** RTS, Respiratory tract secretion; ST, Sequence typing; *cpn60*, 60-KDa chaperonin; *fusA*, elongation factor EF-G; *gltA*, citrate synthase; *pyrG*, CTP synthase, *recA*, homologous recombination factor; *rplB*, 50S ribosomal protein L2; *rpoB*, RNA polymerase subunit B; IMP, Imipenem; MER, Meropenem; DOR, Doripenem; CTZ, Ceftazidime; GEN, Gentamicin; AMK, Amikacin; CIP, Ciprofloxacin; COL, Colistin; *bla*, beta-lactamase; *NDM*, New delhi metallo-beta-lactamase; *mcr-1*, mobilized colistin resistance; *OXA*, Oxacillinase
